# Mismatch of Low Perfusion and High Permeability Predicts Hemorrhagic Transformation Region in Acute Ischemic Stroke Patients Treated with Intra-arterial Thrombolysis

**DOI:** 10.1038/srep27950

**Published:** 2016-06-15

**Authors:** Hui Chen, Nan Liu, Ying Li, Max Wintermark, Alan Jackson, Bing Wu, Zihua Su, Fei Chen, Jun Hu, Yongwei Zhang, Guangming Zhu

**Affiliations:** 1 Department of Neurology, Military General Hospital of Beijing PLA, Beijing, 100700, China; 2 Stanford University, Department of Radiology, Neuroradiology Section, Stanford, CA, USA; 3 Wolfson Molecular Imaging Centre, University of Manchester, Manchester, UK; 4 Department of Radiology, Military General Hospital of Beijing PLA, Beijing, 100700, China; 5 GE Healthcare, Beijing, China; 6 Department of Neurology, Southwest Hospital, Third Military Medical University, Chongqing, 400038, China; 7 Department of Neurology, Changhai Hospital, Second Military Medical University, Shanghai, 200433, China

## Abstract

This study sought to determine whether the permeability related parameter K^trans^, derived from computed tomography perfusion (CTP) imaging, can predict hemorrhagic transformation (HT) in patients with acute ischemic stroke who receive intra-arterial thrombolysis. Data from patients meeting the criterion were examined. CTP was performed and K^trans^ maps were used to assess the permeability values in HT and non-HT regions. A receiver operating characteristic (ROC) curve was calculated, showing the sensitivity and specificity of K^trans^ for predicting HT risk. Composite images were produced to illustrate the spatial correlations among perfusion, permeability changes and HT. This study examined 41 patients. Twenty-six patients had hemorrhagic infarction and 15 had parenchymal hemorrhage. The mean K^trans^ value in HT regions was significantly lower than that in the non-HT regions (0.26 ± 0.21/min vs. 0.78 ± 0.64/min; P < 0.001). The ROC curve analysis identified an optimal cutoff value of 0.334/min for K^trans^ to predict HT risk. Composite images suggested ischemic regions with low permeability, or the mismatch area of low perfusion and high permeability, more likely have HT. HT regions after intra-arterial thrombolysis had lower permeability values on K^trans^ maps. The mismatch area of lower perfusion and higher permeability are more likely to develop HT.

Intracranial hemorrhage is an undesirable complication of reperfusion therapy after acute ischemic stroke (AIS). Numerous clinical risk factors for thrombolysis-related cerebral hemorrhage have been identified, including the type of intervention^1^, stroke severity^2^, post-thrombolysis blood pressure (BP)^3^, concurrent use of antithrombotic agents^4^, and time to symptom onset^4^. The use of multi-modality magnetic resonance imaging (MRI) and computed tomography (CT), especially perfusion imaging, has led to additional predictive parameters for hemorrhagic transformation (HT) after thrombolysis, including the hyperdense middle cerebral artery sign^5^, the volume of hypodensity on admission CT^6^, high pre-treatment volumes of diffusion weighted imaging (DWI) deficits^3^, and very low cerebral blood volume (CBV)^7^.

Changes in blood-brain barrier permeability (BBBP), which are seen in some forms of cerebral insult, are also considered a promising predictor of HT after AIS^8^. There are a number of methods available to assess BBBP, all based on dynamic imaging of contrast agent distribution. These include perfusion CT (CTP)^9^, dynamic susceptibility contrast (DSC) MRI^10^, and dynamic contrast-enhanced MRI using T1 weighted images^11^. Most studies suggest that areas with increasing BBBP values are more likely to proceed to HT after reperfusion therapy^9^,^12^.

The estimation of BBBP is based on pharmacokinetic modeling of contrast agent distribution and is dependent on the effective delivery of the contrast agent to the location being studied. Current pathophysiological theories suggest that the greatest damage to the vessels will occur in areas near a blood clot resulting in a consequent elevation of capillary endothelial permeability. However, where vessels are blocked and contrast agent delivery does not occur, calculated values of permeability will be artifactually reduced.

In this study, we hypothesized that BBBP is low in severely damaged tissue before reperfusion. We examined this hypothesis by testing the prediction that the permeability-related parameter K^trans^, a volume transfer constant^12^ derived from CTP, would 1) predict HT and 2) distinguish between hemorrhagic infarction (HI) and parenchymal hemorrhage (PH) in patients with AIS who receive intra-arterial thrombolysis with tissue plasminogen activator (tPA).

## Methods

### Patients

The clinical and imaging data presented in this study were retrieved from a repository created from data collected as part of standard clinical stroke care at three participating institutions, including Military General Hospital of Beijing PLA, Beijing, Southwest Hospital, Chongqing, and Changhai Hospital, Shanghai. Only completely anonymized data were contributed to the repository.

Authors confirm that all experiments were performed in accordance with relevant guidelines and regulations. The institutional review boards of Military General Hospital of Beijing PLA, Southwest Hospital, and Changhai Hospital approved the collection, analysis of data from the repository and all experimental protocols, including any other relevant details. The methods were carried out in accordance with the approved guidelines. The written informed consent was obtained from all subjects.

**Figure 1:**
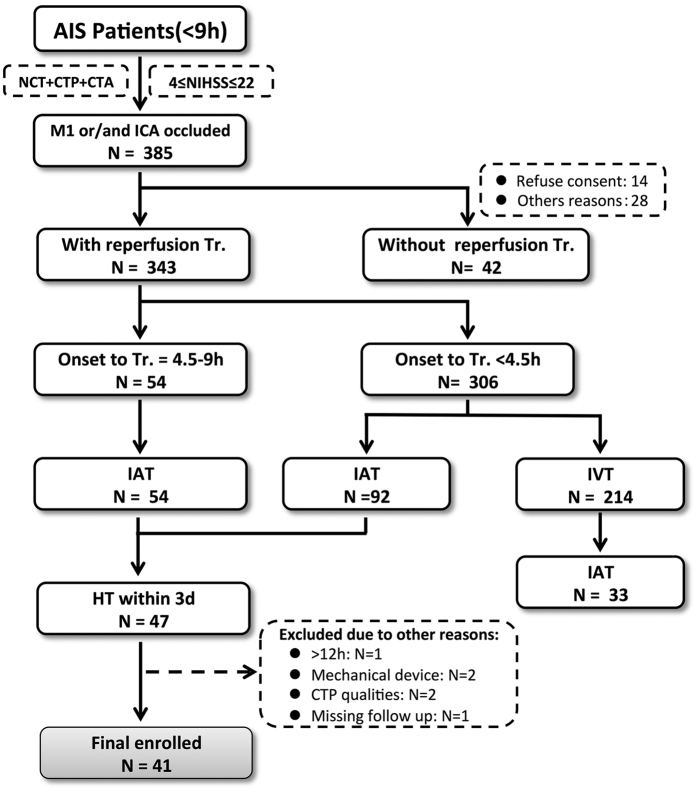
Flow chart outlining patient selection. AIS: acute ischemic stroke, NCT: non-contrast computed tomography, CTP: CT perfusion, CTA: CT angiography, NIHSS: National Institutes of Health stroke scale, M1: the M1 segment of the middle cerebral artery, ICA: internal carotid artery, Tr.: treatment, IAT: Intra-arterial thrombolysis, IVT: Intra-venous thrombolysis, HT: hemorrhagic transformation.

We retrospectively identified all consecutive patients admitted to these institutions with signs and symptoms suggestive of hemispheric stroke between January 2011 and January 2014 who met the following inclusion criteria: 1) AIS with occlusion of the M1 segment of the middle cerebral artery (MCA), the internal carotid artery (ICA), or both and a National Institutes of Health stroke scale (NIHSS) score between 4 and 22 upon admission; 2) completion of a stroke CT imaging work-up including non-contrast CT, CT angiography (CTA), and CTP at admission; 3) treatment involving intra-arterial thrombolysis with tPA, with less than 12 h from stroke onset to the end of thrombolysis; 4) presence of HT at 3 days post-ictus, demonstrated on a gradient-echo T2-weighted MR image; and 5) availability of MR or CT at 2 weeks post-ictus to assess the final infarct volume. Patients were excluded if: 1) IV tPA treatment was administered prior to angiography or 2) patients received reperfusion treatment using mechanical thrombolytic devices. A flow chart outlining the patient selection process is presented in Fig. 1.

The following demographic and clinical variables were recorded: age, gender, medical history, vascular risk factors, the time from symptom onset to treatment, NIHSS upon admission, and modified Rankin scale (mRS) score at 90 days post-ictus. The 90-day outcomes were assessed in an outpatient clinic or over the telephone. A mRS score of 2 or lower was considered a good clinical outcome, and an mRS of 6 indicates death. The stroke mechanism was subtyped using the Trial of Org10172 in Acute Stroke Treatment classification and diagnosed by the consensus of two stroke neurologists.

### Image processing and interpretation

The CTP, CTA, digital subtraction angiography, and MR imaging protocols used are described in our previous study^13^. The CTP data were analyzed using dedicated software (MIStar: Apollo Medical Imaging, Melbourne, Australia). This software applies a singular value decomposition with delay- and dispersion-correction to calculate parametric perfusion maps and automatically measure the volumes of the infarct and penumbra, which are defined as areas with a delay time >3 s and cerebral blood flow (CBF) <30% of the contralateral CBF^14^, respectively.

**Figure 2:**
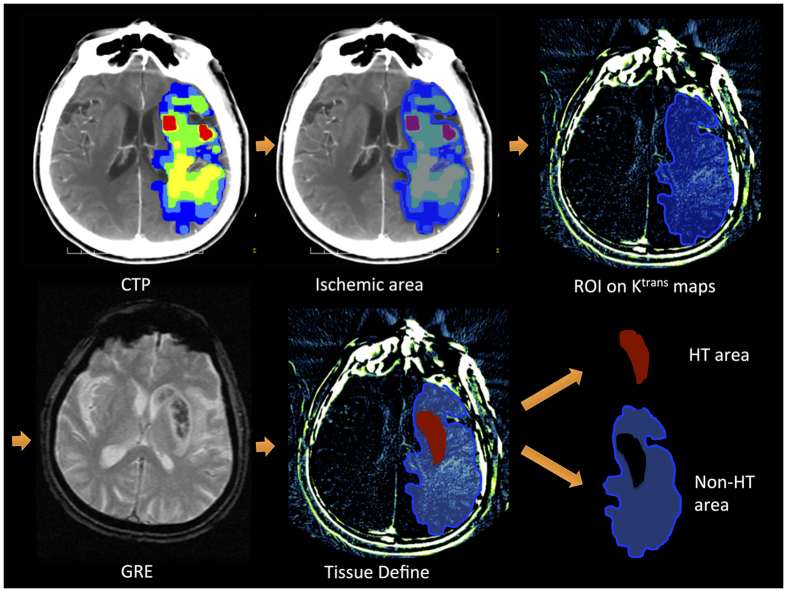
The protocol used to define the regions of interest (ROIs) for both hemorrhagic transformation (HT) and Non-HT areas on K^trans^ maps. **Step 1:** The perfusion computed tomography (CTP) output images were used to draw the distribution of the ischemic area manually. **Step 2:** The ischemic area was overlapped on the K^trans^ map to determine the ROIs, and all permeability values were restricted to this area. **Step 3:** ROIs were divided into HT areas and Non-HT areas, corresponding to the HT regions on the gradient recalled echo (GRE) T2-weighted magnetic resonance image.

Parametric maps of K^trans^ were calculated from the CTP data using prototype software (OmniKinetics, GE Healthcare China)^15^ under a Patlak model^16^ fitted to the data recorded during the first passage of the contrast agent. Advantages of this approach include simplicity and improved stability compared to alternative curve fitting approaches, especially in the presence of low levels of contrast agent leakage. Parametric maps of K^trans^ were loaded into ImageJ software version 1.47 for Mac OS (Wayne Rasband, National Institutes of Health, USA), two neurologists (H.C. and Y.L.) manually defined the regions of interest (ROIs), and the mean K^trans^ values were derived for each ROI. The K^trans^ values in all ROIs were measured on 3 separate occasions by each neurologist to calculate the intra- and inter-observer agreement. The protocol used to define the ROIs is illustrated in Fig. 2.

In the first analysis, the 41 patients were divided into three groups based on the gradient-echo T2-weighted^17^ images performed on day 3, using the European Cooperative Acute Stroke Study^18^ classification of hemorrhagic infarct: 1) no HT; 2) HI (hemorrhagic infarction; petechial HT) and 3) PH (parenchymal hemorrhage; HT with or without mass effect). We compared the K^trans^ values in cerebral regions with and without HT, and we compared the K^trans^ values between the two HT subtypes (HI and PH). These analyses were performed for the parenchymal tissue lying within the ischemic area defined on CTP.

In the second analysis, the spatial distribution of HT, the permeability changes and perfusion status were assessed according to the Alberta Stroke Program Early CT Score (ASPECTS) system. There are 10 ASPECTS regions: caudate I, lenticular (L), internal capsule (IC), insular ribbon (I), and M1-M6. Based on the admission CTP and follow up gradient-echo T2-weighted sequence, all ASPECTS regions were divided into four categories: 1) ischemic tissue without HT (Non-HT); 2) ischemic tissue with HI (HI); 3) ischemic tissue with PH (PH); and 4) normal tissue without ischemia (normal). There were no regions where CTP demonstrated HT in the absence of ischemia. The number of regions in each of the above categories was counted.

Composite images were produced to demonstrate the spatial correlations among permeability changes, perfusion status and HT. For each patient, HT lesions were extracted from gradient-echo T2-weighted images. HT areas were coded and a parametric map showing the relative risk of HT was produced. This probability map was superimposed onto the corresponding T1-weighted anatomical MR image. The procedure was repeated for the Tmax map to display the distribution of low perfusion, and for the K^trans^ maps to display the distribution of BBB disruption. Tmax maps DT + 10, DT + 8, DT + 6 meant the delay time >10 s, 8 s and 6 s, respectively. The composite maps allow direct visualization of the probability of HT, perfusion status and change in BBBP within the each brain area in the study group.

### Statistical analyses

The baseline characteristics between the HI and PH groups were compared. Age and systolic and diastolic BP upon admission were compared using Student’s t-tests. The time from symptom onset to imaging, NIHSS on admission, ASPECTS on NCT, collateral flow score, percentage of carotid artery stenosis, CTP infarct core, CTP ischemic penumbra, CTP total ischemic volumes, permeability values of HT regions, permeability values of Non-HT regions, final ischemic volume, and 90-day mRS (≤2 vs. > 2) were compared using Wilcoxon Rank Sum Tests. Pearson’s Chi-squared test was used to analyze the relationship between stroke subtypes and sites of occlusion. Gender, hypertension, diabetes, hyperlipidemia, atrial fibrillation, current smoking, and the use of statins upon admission were compared using Pearson’s Chi-squared test with Yates’ continuity correction. Coronary heart disease and recanalization were compared using Fisher’s exact test. The mean permeability values of HT regions and non-HT regions for all 41 patients were compared using Wilcoxon Rank Sum Tests, and a receiver operating characteristic (ROC) curve was calculated to show the sensitivity and specificity of the K^trans^ value for predicting HT risk. Intra-class correlation coefficients were used to analyze the intra- and inter-observer agreement for assessing the permeability values of HT and non-HT regions. All ischemic ASPECTS regions of the 41 patients were divided into 4 categories: HI, PH, Non-HT, and Normal. The mean K^trans^ values among these 4 groups were compared using Kruskal-Wallis Rank Sum Tests. All statistical analyses were carried out using SAS version 9.3 (SAS Institute, Cary, NC). A value of *P* < 0.05 was considered statistically significant.

## Results

Of the 41 patients who met our criteria, HI was found in 26 patients (63.4%) and PH in 15 (36.6%). NIHSS scores on admission showed a trend towards higher scores in the PH group, but this failed to reach statistical significance (P = 0.080). No differences between the HI and PH groups were observed in any other baseline characteristics (Table 1).

**Figure 3:**
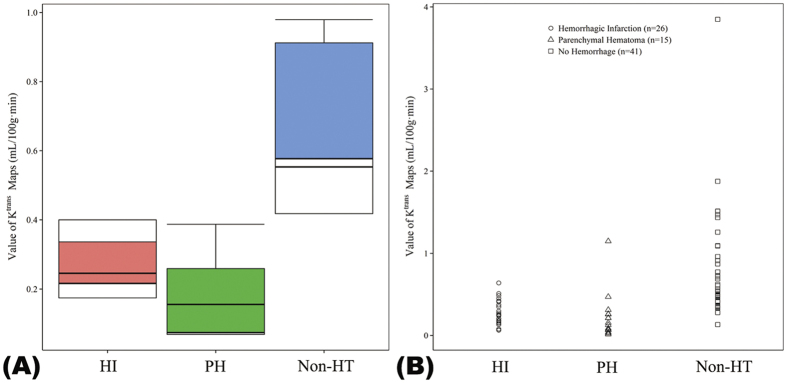
Box plots and distribution of K^trans^ values in different regions. (**A**) Box plots of K^trans^ values in hemorrhagic infarction (HI), parenchymal hemorrhage (PH) and non-hemorrhagic transformation (Non-HT) regions. In each box, the median, 95% confidence interval (CI), and first and third quartile values are illustrated. (**B**) The distribution of K^trans^ values grouped into HI, PH, and Non-HT regions.

The K^trans^ value of the HT regions showed a trend toward higher values in the HI group than the PH group, but the difference did not reach statistical significance (0.28 ± 0.15/min vs. 0.23 ± 0.28/min; P = 0.088; Table 1 and Fig. 3). The K^trans^ values of Non-HT regions were similar in both the HI and PH groups (0.79 ± 0.44 and 0.76 ± 0.91/min; P = 0.199).

**Figure 4:**
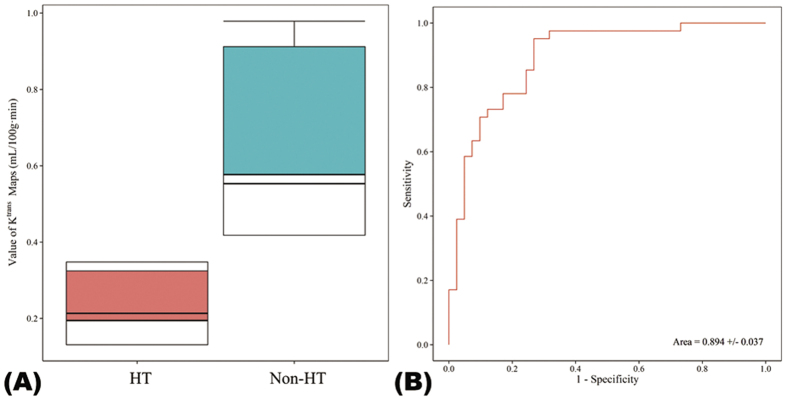
Box plots of K^trans^ values of hemorrhagic transformation (HT) and Non-HT regions and the K^trans^ receiver operating characteristic (ROC) curve. (**A)** Box plots of K^trans^ values in HT and Non-HT regions. In each box, the median, 95% confidence interval (CI), and first and third quartile values are illustrated. (**B)** An optimal cutoff value of K^trans^ to predict HT was identified using the ROC curve analysis.

Across all 41 patients, the mean K^trans^ value of the HT regions (0.26 ± 0.21/min) was significantly lower than that in the Non-HT regions (0.78 ± 0.64/min; P < 0.001). Compared to regions without HT, significantly lower K^trans^ values were seen in HT regions in both the HI and PH groups (P < 0.001; Table 1 and Fig. 4). An ROC curve analysis identified an optimal cutoff value for K^trans^ of 0.334/min, producing a mean area under the curve of 0.894 ± 0.037. The sensitivity and specificity at this threshold were 95.1% and 73.2%, respectively (Fig. 4). The intra-observer agreement between the two neurologists was good. In HT regions, both intra-class correlation coefficients were 0.876 (95% CI 0.792–0.930, P < 0.0001), corresponding to 0.985 (95% CI 0.976–0.992, P < 0.001) and 0.994 (95% CI 0.991–0.997, P < 0.0001), respectively, in non-HT regions. The inter-observer agreement between two neurologists was 0.957 (95% CI 0.920–0.977, P < 0.0001) in HT regions and 0.972 (95% CI 0.948–0.985, P < 0.0001) in non-HT regions.

Of all 410 ASPECTS regions, 330 were ischemic; 30 of these developed HI and 25 PH. The majority of HI (63.3%; 19/30) and PH (44%; 11/25) occurred in the lentiform nucleus. Another region that had a high risk of HT was the insular cortex, representing 20% (6/30) of HI regions and 20% (5/25) of PH regions.

**Figure 5:**
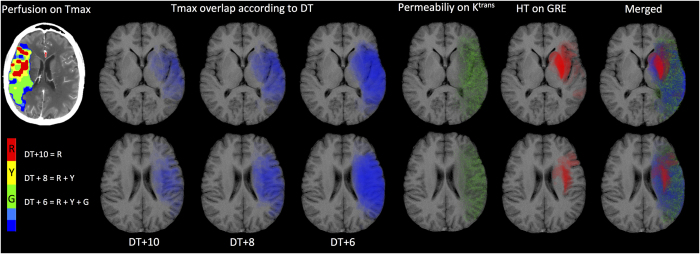
The spatial distribution of hemorrhagic transformation (HT), permeability, and perfusion change. **Column One:** Perfusion maps on Tmax. DT + 10 indicates the most severe ischemic area (DT >10 s), which are the red areas on Tmax. DT + 8 indicates the moderate to severe ischemic area (DT >8s), which are the red and yellow on Tmax. DT + 6 indicates the mild to severe ischemic area (DT >6 s), which are the red, yellow and green on Tmax. **Column Two to Four:** Overlapped ischemic area on T1-based Alberta Stroke Program Early CT Score (ASPECTS) system of classification. The blue shapes indicate ischemic lesions which didn’t show specific correction with HT area, no matter DT was >10 s, >8 s, or >6 s. **Column Five:** The green dots from overlapped permeability changes indicate the blood brain barrier (BBB) disruption detected by K^trans^ maps. The lenticular region did not demonstrate increases in BBB permeability compared with other areas. **Column Six:** The red shapes indicate over lapped HT lesions and the depth of red represents the frequency of HT occurrence. The lenticular and insula regions had the greatest risk of HT in this study. **Column Seven:** The merged picture of perfusion (DT >6 s), permeability on K^trans^ maps and HT indicates the specific spatial correlation between HT and the mismatch of low perfusion and high permeability.

The spatial distribution of perfusion, permeability, HT, and their merged picture are illustrated in composite ASPECTS images (Fig. 5). The lentiform nucleus had the highest risk of HT, thus colored the darkest red, corresponding to an area where the cumulative permeability value on the K^trans^ maps was lower, within those areas where the perfusion was lower (blue) and permeability value was higher (green). In another word, from the merged map, the mismatched area between low perfusion (blue) and high permeability (green) is exactly the HT red area (Fig. 5).

## Discussion

HT is common in AIS patients following intra-arterial thrombolysis, and the clinical outcome of PH is poor. A number of characteristics have been associated with an increased risk of HT, including stroke severity^2^, time to reperfusion^19^, thrombolytic protocol violations, tPA treatment^2^, white matter disease burden^20^, aspirin^21^, and heparin use^22^. In addition, a number of imaging parameters have been associated with an increased risk of HT, including DWI lesion volume, severe hypo-perfusion measured by prolonged contrast arrival times and very low values of regional CBV^7^.

HT in AIS is associated with the loss of BBB integrity^23^. In particular, there is loss of adherence in the tight junctions between cerebral capillary endothelial cells, which appears to reflect a loss of occludin and redistribution of claudin-5 proteins mediated by astrocytes^24^. These changes allow abnormal leakage of proteins and other molecules from the plasma into the extra-vascular space^8^. Both acute ischemia and tPA therapy contribute to this BBB damage^25^. Dynamic contrast-enhanced imaging techniques provide an attractive approach for the evaluation of BBB integrity in a clinical setting. Conventional radiological contrast agents do not cross the intact BBB; therefore, measurement of contrast agent leakage is an appropriate surrogate indicator of endothelial tight junction failure. A number of previous studies, using both dynamic contrast-enhanced MRI and DSC MRI, have examined the relationship between HT and changes in BBB permeability in patients with AIS^10^,^26^. Unfortunately, the results of these studies have been contradictory, and the clinical threshold value of BBBP for predicting the likelihood of HT remains unclear^27^. One significant problem associated with the use of dynamic contrast-enhanced MRI is that MRI is not used as a first-line investigation in the acute stroke setting in most centers. In addition, dynamic contrast enhanced MR sequences add significant imaging time to standard diagnostic protocols. This means that the estimation of BBBP from MRI may require additional diagnostic investigation at a critical therapeutic time. A further major consideration is the significant variation that can be seen in dynamic contrast-enhanced MRI and, to a lesser extent, in DSC MRI obtained with different acquisition protocols and on different imaging systems. This can introduce very significant variation when estimating the measurement of parameters such as K^trans^, making multicenter implementation problematic^28^.

In contrast, CTP fits into the clinical management pathway for AIS well. CT is the initial diagnostic modality in the vast majority of centers, and the addition of CTP requires only a short period of additional imaging. CTP data are extremely robust and do not suffer from the acquisition system-dependent variations seen with MR data. Technical validation of multicenter implementation is relatively straightforward, which has led to significant growth in the use of CTP for both ischemic and oncological brain diseases^29^. CTP data can be analyzed using the same range of semi-quantitative and pharmacokinetic metrics described for use with dynamic MR data; the application of simple models, such as the Patlak model employed in this study, provides a rapid, simple, and robust acquisition and analytical approach^16^,^30^.

In current study, either severe or moderate ischemic area on Tmax didn’t show the specific spatial correlation with HT. And HT regions after intra-arterial thrombolysis had lower permeability values on K^trans^ maps. But only using low values on K^trans^ maps to predict HT is still impossible, because normal tissues also had lower permeability values. This suggested the need to use both perfusion and permeability variables together. As illustrated by Fig 5, the risk of HT is highest in the mismatch area between low perfusion and high permeability, or in another word, those ischemic regions with lower estimated K^trans^ values. This contradicts the results a number of previous studies that found evidence of higher permeability in infarcts that subsequently progressed to HT^11^,^27^,^31^,^32^. Although this major difference appears initially counterintuitive, the apparent discrepancy between this and previous studies likely reflects methodological differences, which are discussed below. Furthermore, we believe that it supports our hypothesis that HT is more likely to occur in areas with the most severe hypo-perfusion and ischemic injury.

In the normal brain, the intact BBB prevents leakage of the contrast agent from the intravascular space, such that K^trans^ cannot be measured. There is no doubt from this and previous studies that areas of acute ischemia in AIS can demonstrate significant elevation of BBBP, keeping with the pathological and histological findings discussed above. Moreover, a higher mean K^trans^ value within the infarcted area is associated with an increased risk of HT^32^. However, significant heterogeneity exists within an ischemic area, including both areas of relative blood flow preservation and areas of continued severe hypo-perfusion even following reperfusion. The contrast transfer coefficient, K^trans^, is widely used as a direct surrogate of cerebral capillary endothelial permeability. However, trans-endothelial leakage can occur only where there is vascular delivery of contrast agent. Where blood flow is low, contrast agent delivery is severely limited and K^trans^ becomes flow-dependent, because the amount of contrast agent leaking depends on the amount delivered to the tissue rather than its ability to leak through the endothelium^33^. Previous studies have focused on mean K^trans^ values derived from within the entire infarct volume as a predictive feature for HT. In the present study, we have focused specifically on areas of the brain that subsequently develop HT. We have shown that, although HT may be associated with infarcts that show generally high BBBP, HT occurs in areas of the infarct where K^trans^ measurements are low. We identified an upper K^trans^ threshold of 0.334/min that predicted HT with 95% sensitivity and 73% specificity in our study population. The relationship among reduced K^trans^ and a greater risk of HT after reperfusion was also supported by the anatomical dichotomy between HT risk and K^trans^ elevation demonstrated on the combination maps (Fig. 3). But, importantly, both normal and severe hypo-perfusion regions had low K^trans^ value, which meant perfusion variables were still needed to define the ischemic borderline within where K^trans^ values were meaningful. This was the main reason we use the mismatch of Tmax and K^trans^ maps.

To our surprise, we didn’t find statistical difference on the severity of hypertension between the groups in our study. Though uncontrolled hypertension may have important impacts on clinical outcome in long term, the hypertension in acute stage may have some protection for cerebral perfusion as well as be a risk factor for hemorrhagic transformation, liking a dual-edged sword. This bidirectional effect may explain the negative result.

We acknowledge a number of limitations to our study. First, it is a retrospective study involving a limited number of cases. Second, we did not include AIS patients who did not develop HT after reperfusion, which could lead to bias. Third, the use of gradient-echo T2-weighted images to determine HT may also have some limitations, and susceptibility-weighed imaging may have yielded greater sensitivity. Also, bias may arise due to the different methods which the patients were followed with.

In conclusion, we demonstrated that the risk of HT for acute proximal arterial occlusion after intra-arterial reperfusion could be predicted using K^trans^ maps derived from standard first-pass CTP and Tmax maps. In contrast to previous studies, we showed HT regions after intra-arterial thrombolysis had lower permeability values on K^trans^ maps, and mismatch areas of low perfusion and high permeability are more likely to develop HT. Further studies are needed to examine the potential utility of this technique for influencing decision making regarding the treatment of patients with AIS.

## Additional Information

**How to cite this article**: Chen, H. *et al*. Mismatch of Low Perfusion and High Permeability Predicts Hemorrhagic Transformation Region in Acute Ischemic Stroke Patients Treated with Intra-arterial Thrombolysis. *Sci. Rep.* **6**, 27950; doi: 10.1038/srep27950 (2016).

## Figures and Tables

**Table 1:** Patient characteristics by HT subtype.

Characteristics		All patients N = 41	HI N = 26	PH N = 15	P Value
Clinical data					
Female patients^★^		16	11	5	0.814
Age (yr)^▲^		64.37 ± 12.45	63.27 ± 13.38	66.27 ± 10.83	0.465
Time from onset to treatment (h)^△^		5.93 ± 3.59	5.64 ± 3.74	6.43 ± 3.39	0.408
NIHSS on admission^△^		15.73 ± 4.63	14.98 ± 4.19	17.01 ± 5.29	0.080
Hypertension^★^		23	13	10	0.478
Diabetes^★^		7	5	2	0.958
Hyperlipidemia^★^		7	3	4	0.418
Atrial fibrillation^★^		10	6	4	1.000
Coronary heart disease^#^		5	3	2	1.000
Currently smoking^★^		21	13	8	1.000
Current statin use^★^		9	5	4	0.871
Systolic pressure on admission (mmHg)^▲^		143.93 ± 25.29	142.12 ± 24.85	147.07 ± 26.60	0.553
Diastolic pressure on admission (mmHg)^▲^		73.98 ± 14.20	72.46 ± 12.25	76.60 ± 17.23	0.376
Type of stroke^☆^					
Cardioembolism		10	7	3	0.687
Large-artery atherosclerosis		17	9	8	
Other		6	4	2	
Unknown		8	6	2	
Imaging data					
ASPECT score^△^		7.44 ± 1.58	7.54 ± 1.56	7.27 ± 1.67	0.599
Collateral flow score^△^		1.80 ± 1.03	1.62 ± 1.06	2.13 ± 0.92	0.125
Percentage of carotid artery stenosis (%)^△^		45.00 ± 47.42	45.77 ± 48.53	43.67 ± 47.07	1.000
Infarct core on CTP (ml)^△^		26.15 ± 19.01	26.65 ± 16.79	25.29 ± 22.97	0.495
Ischemic penumbra on CTP (ml)^△^		44.07 ± 20.90	41.72 ± 21.46	48.15 ± 19.94	0.165
Total ischemic volume on CTP (ml)^△^		70.22 ± 23.62	68.37 ± 18.05	73.43 ± 31.54	0.779
Permeability value of HT regions (/min)^△^		0.26 ± 0.21	0.28 ± 0.15	0.23 ± 0.28	0.088
Permeability value of Non-HT regions(/min)^△^		0.78 ± 0.64	0.79 ± 0.44	0.76 ± 0.91	0.199
Occluded artery^☆^	M1	24	16	8	0.456
	ICA	7	3	4	
	M1 + ICA	10	7	3	
Outcome					
Successful recanalization^#^		35	23	12	0.780
Final ischemic volume (ml)^△^		46.42 ± 37.26	38.04 ± 28.23	60.96 ± 46.75	0.155
mRS score at 90 days post stroke^△^		3.27 ± 1.80	3.04 ± 1.71	3.67 ± 1.95	0.357

Data are presented as the mean ± standard deviation or the count (N). HI: hemorrhagic infarction, PH: parenchymal hemorrhage, NIHSS: National Institutes of Health stroke scale, ASPECT score: Alberta Stroke Program Early CT score, CTP: perfusion computed tomography, HT: hemorrhagic transformation, M1: the M1 segment of the middle cerebral artery, ICA: internal carotid artery, mRS: modified Rankin scale. ^▴^Student’s t-Test; ^▵^Wilcoxon Rank Sum Test; ^☆^Pearson’s Chi-squared test; ^★^Pearson’s Chi-squared test with Yates’ continuity correction; ^#^Fisher’s exact test.
